# The phylogenetic status of typical Chinese native pigs: analyzed by Asian and European pig mitochondrial genome sequences

**DOI:** 10.1186/2049-1891-4-9

**Published:** 2013-03-08

**Authors:** Guanghui Yu, Hai Xiang, Jikun Wang, Xingbo Zhao

**Affiliations:** 1 National Engineering Laboratory for Animal Breeding, Ministry of Agricultural Key Laboratory of Animal Genetics, Breeding and Reproduction, State Key Laboratory for Agribiotechnology, College of Animal Science and Technology, China Agricultural University, Beijing, 100193, China

**Keywords:** Mitochondrial DNA, Origin and evolution, Phylogenetic analysis, Pig

## Abstract

China is one of the most diverse countries, which have developed 88 indigenous pig breeds. Several studies showed that pigs were independently domesticated in multiple regions of the world. The purpose of this study was to investigate the origin and evolution of Chinese pigs using complete mitochondrial genomic sequences (mtDNA) from Asian and European domestic pigs and wild boars. Thirty primer pairs were designed to determine the mtDNA sequences of Xiang pig, Large White, Lantang, Jinhua and Pietrain. The phylogenetic status of Chinese native pigs was investigated by comparing the mtDNA sequences of complete coding regions and D-loop regions respectively amongst Asian breeds, European breeds and wild boars. The analyzed results by two cluster methods contributed to the same conclusion that all pigs were classified into two major groups, European clade and Asian clade. It revealed that Chinese pigs were only recently diverged from each other and distinctly different from European pigs. Berkshire was clustered with Asian pigs and Chinese pigs were involved in the development of Berkshire breeding. The Malaysian wild boar had distant genetic relationship with European and Asian pigs. Jinhua and Lanyu pigs had more nucleotide diversity with Chinese pigs although they all belonged to the Asian major clade. Chinese domestic pigs were clustered with wild boars in Yangtze River region and South China.

## Background

Pig (*Sus scrofa*) is one of the most important economic animals, and it distributes widely in the world from cold belt to tropical zone. Although it has the raising history more than 8,000 years [1], the origin and evolution of Chinese domestic pigs is still uncertain and there are several controversial viewpoints on this scientific problem. Some people think the pig domestication have even along the Yellow River and Inner Mongolia [2]. While the available zoo archaeological evidence has been interpreted to indicate that domestic pigs were prevalent in both northern and southern China [3] and widespread wild boar populations that have not contributed maternal genetic material to modern domestic stocks [1].

Mitochondrial DNA has the typical characteristics: 1. Evolution is more diverse than nuclear DNA [4-7]. 2. Evolution of mammalian mtDNA occurs primarily as single base pair substitutions, with only infrequent major sequence rearrangements [8] 3. The mtDNA is maternally inherited, haploid and non-recombining [9]. So mtDNA is one of the most popular markers used in determining relationships among individuals within species and among closely related species with recent times of divergence [4,10]. The D-loop region of mtDNA is known to be more variable in sequence than other regions [11]. For evolution research, a number of studies of the D-loop region sequence mutation in mtDNA have also been investigated [12-15]. And thus also has been frequently used for phylogenetic analysis of closely related groups, especially for determining intra-specific phylogenies [16].

Multiple origins have been revealed to be a common phenomenon in domestic animals such as cattle, goats, chicken, and horses [7,17-19]. Several studies also have shown that pigs were independently domesticated in various parts of the world [20-22]. However, the most popular researches support the independent origin of domestic pigs in Europe and Asia as the time of divergence between European and Asian pig mitochondrial mtDNAs was long before the time of possible pig domestication [20,23]. Recent studies have revealed a schematic profile concerning the origin of wild boars [24] and their dispersal and domestication across Eurasia, as well as the Neolithic expansion in Island South East Asia and Oceania by analyzing the mtDNA D-loop sequences of worldwide wild boars, domestic pigs, and ancient specimens [21,25,26]. Pigs indigenous to China, Korea and Japan, including Wild Boars sampled from the area, are closely related and different from European pigs in their maternal lineages. An almost equal distance was found between the European Wild Boar sample and European-type breeds (0.0168) or Asian-type breeds (0.0181), suggesting that certain European Wild Boars may be ancestors of both European and Asian pigs [27]. This finding contrasts with the report of independent domestication of Asian and European pigs from their respective Wild Boars [20].

China is one of the most early countries originally feed pigs. And the extremes of climate and geography have contributed to the development of more than 80 indigenous breeds, many of which have special and unique characteristics. Many breeds are considered rare, have a small population size, and are under increasing pressure from the introgression of modern commercial breeds. This makes investigations of both population structure and genetic diversity increasingly important [28]. The purpose of this study was to sequence the complete mtDNA of three Chinese pig breeds and two European pig breeds. Other pigs and wild boars mitochondrial genomic sequences submitted to GenBank were also invited in this study. Population phylogenomic analysis was conducted in domestic pigs and wild boars by screening a phylogenetic tree of the mtDNA complete coding region sequences and compared to the D-loop sequences to investigate the origination migration and evolution of Chinese pig populations.

## Methods

### Sample collection, DNA extraction and complete mtDNA genome sequencing

A total of 3 Chinese pig breeds (Xiang pig, Lantang and Jinhua) and 2 European pig breeds (Large White and Pietrain) were invited in this study for the complete mtDNA genomic sequences and used together with the other domestic pig and wild boar sequences download from the GenBank to analyze the origin and evolution of Chinese pigs. All of the pig breeds, geographic classification and GenBank Accession Numbers were showed in Table 1. Geographic definition of regions was based on a former research [3].

**Table 1:** Pig breeds and their geographic definitions of regions

**Geographic definitions**	**Pig breeds**
North East Asia (NEA)	Min (AF486864.1), WB-Korea (AY574047.1), Jeju native pig (AY879785.1), Korean native pig (AY879794.1), WB-China northeast (EU333163.1), WB-Japan (AB015085.1)
Yellow River Valley (YR)	Bamei (EF545583.1), Yimeng Black (AF486868.1), Huzu (EF545588.1)
Yangtze River Region (YZ)	Aba (EF545578.1), Erhualian (AF486861.1), Jiangquhai (AF486872.1), Qingping (AF486865.1), Rongchang (AF486860.1), Tongcheng (AF486862.1), Wannanhua (AF486873.1), WB-Jiangxi (EF545579.1), Yushan Black (AF486871.1), Zang (AF486856.1), Zhong Meishan (AF486855.1), Jinhua (KC469586), Xiang Pig (KC250273), Bihu (EF545591.1), Kele (EF536857.1), Taoyuan (AM040653.1), Wei (EF545577.1)
Mekong Region (MR)	Diannan Short-ear (AF486869.1), WB-Malaysia (EF545592.1), WB-Vietnam (EF545584.1), WB-Yunnan (EF545573.1), Banna mini (GQ220328.1), Dahe (GQ220329.1), Thailand indigenous pig (FM244493.1)
South China (SC)	Dahuabai (AF486870.1), Ningxiang (AF486857.1), Lanyu (DQ518915.2), WB-Fujian (EF545569.1), WB-Hainan (EF545572.1), Wuzhishan (AF486867.1), Lantang (KC250274)
European Country (EU)	Berkshire (AY574045.1), Duroc (AY337045.1), Hampshire (AY574046.1), Landrace (AF034253.1), WB-Italian (AF304201.1), Pietrain (KC469587), Large White (KC250275), Iberian (FJ236994.1), WB-European (FJ237000.1)

The blood of Jinhua and Pietrain and cells of Lantang, Xiang pig and Large White were collected. And the genomic DNA was extracted by standard phenol-chloroform method. A primer pair was used to amplify the D-loop region of pig mtDNA, forward: 5^′^-AGGAGACTAACTCCGCCAT-3^′^; reverse: R: 5^′^-CGCGGATACTTGCATGTGT-3^′^. Another 29 primer pairs were used to amplify the complete coding region sequences (Table 2). Routine PCRs were conducted in gradient thermal recyclers. The reaction system was conducted in a 25 μL volume containing 2.5 μL of 10 × buffer (with Mg^2+^), 0.25 mmol/L dNTPs, 0.2 μmol/L each primer, 2 U Taq DNA polymerase and 50 ng gemomic DNA. The reaction began with an initial denaturation at 95°C for 5 min, after that, followed by 30 cycles of denaturation at 95°C for 30 s, annealing at gradients 50–65°C for 30 s, and extension at 72°C for 30 s. The last step was a 5 min final extension period at 72°C. The PCR products were then analyzed by 1% to 1.5% agarose gel electrophoresis, and then gel-extracted and purified for sequencing. Sequences were edited by using the DNASTAR software (DNAstar Inc. Madison, Wisconsin, USA). And the finished data were deposited in NCBI GenBank with accession numbers Xiang Pig (KC250273), Lantang (KC250274), Large White (KC250275), Jinhua (KC469586) and Pietrain (KC469587).

**Table 2:** Primer pairs for pig complete mitochondrial DNA sequences

**Primers NO.**	**Primers sequences 5**^′^**-3**^′^**(Forward/Reverse)**
1	ACTAAGTCAATGCCTATTCTG/CAAATGTATGAAACCTCAG
2	CTACACAATAACCTCCCATA/TGGCACGAGATTTACCAACT
3	GCTCATAACGCCTTGCTC/ATTCTTTCATCTTTCCCTT
4	CACCTAGAAGATCCCACA/ACAACCAGCTATCACCAG
5	CCGTAAGGGAAAGATGAAAG/TATGGTTATTTTGACTGGT
6	CCGTGCAAAGGTAGCATA/CCAACATCGAGGTCGTAA
7	TGGGGTGACCTCGGAGTAC/AATATGGCGAAAGGTCCGG
8	CGAGCAGTAGCCCAAACA/GGTCGTATCGGAATCGTG
9	GTATCAGGCTTTAACGTAGA/TGGTAATACTGCTGTCATTC
10	CACAGAAGCAGCCACAAA/ATGGGATAGGGATAAAGT
11	ACATAGGATGAATGACAGC/TGGTGGAAGTAGTCAGAAAC
12	GCACTGCCTTGAGCCTAC/GTGTTCAGGTTGCGGTCT
13	CTGACTCGTACCGCTAATA/CTGACTCGTACCGCTAATA
14	CACTTTGTAATCATATTCGTAG/TAGTTGGAAAGGGTAAGC
15	TTCATCTCACTAACAGCAG/TTGAGTTCGGTTGATTCTG
16	GCTTCATGCCCATTGTAC/TTATAGCGGAATCCTGTG
17	GCAAGCCCAGAATCAACCG/CGAGGAGGATTGAGGTGTT
18	ATACCACATAGTAAACCCAA/CCTGTAGCCACAAAGAAA
19	CTAAACACCTCAATCCTCC/TTGGACGTAATCGGTACCG
20	CCTTGCAGGGTTACTTAT/TTCGGGTTGTGGTTTCTT
21	CGGTACCGATTACGTCCAA/CCGATTAGATTGATGGATG
22	ACCAGCTCTATCTGCTTA/GAGGCTTTGATGTTGTTA
23	ATGATGACTAATAGCAAGCC/GGGATGTAGTCCGAATTG
24	CATCGGAGACATTGGATT/AGTTGGCTTGAAGTTGAG
25	CCTACTCCTAGCTGCAGCAG/ATTATGGAGATTACTCGTGG
26	TCCGCATCATCATTACTA/TTTATGGTGGACTTGGGT
27	TAATTACCACGAGTAATCTC/TTCTACGAGGTCTGTTCCG
28	GGAGCATCCATATTCTTT/GGTGTAGTTGTCTGGGTCT
29	TCGTAGAATGAATCTGAGG/GGTGATACGCATGTTGACTG

### Phylogenetic analysis

All of the sequences mentioned above were divided into two groups, one was by the D-loop region, and the other was by the complete coding region of mtDNA sequences. The D-loop region was assembled by both overlapping forward and reverse sequencing products. The tandem repeat motif ‘CGTGCGTACA’ was not included in the analysis because the number of repeats was variable within individuals, indicating a high degree of heteroplasmy [29] and thus the repeat itself is not phylogenetically informative. So the D-loop region was 1,045 bp and the complete coding region was 15,435 bp, which used the Large White as the reference sequence. The African warthog (*Phacochoerus africanus*) (NC_008830.1) was used as outgroup because it is well known to be distinct from Eurasian wild boars and has frequently been used in previous phylogenetic studies of pigs [21,30]. All sequences of mtDNA D-loop and complete coding region were aligned using algorithm MUSCLE [31] in MEGA 5 [32] to generate a continuous sequence for each animal. The results were exported and converted into a FASTA format. And then the best fitted model was found by MEGA 5, the complete coding region group used the model of HKY + G, the D-loop group used the model of HKY + I + G. The data were converted into BEAST XML format by BAEUTi 1.7.4 for Bayesian calculation. The parameters were set as: A. selection the counterpart model; B. Yule process for the tree prior superposition; C. the MCMC chain was set to 10,000,000. The calculation was carried out by the software of BEAST 1.7.4 [33]. And TreeAnnotater 1.7.4 was used to summarize the calculation result and find the best supporting phylogenetic tree. Then the tree was depicted using Figure Tree 1.4.0. The nucleotide diversity (π) for the geographic definition breeds were estimated using DnaSP 5.10 [34]. The polymorphisms in the analyzed segments and the pairwise mismatch distribution between different geographic group classifications were obtained using the Arlequin 3.5 computer package [35].

## Results

**Figure 1:**
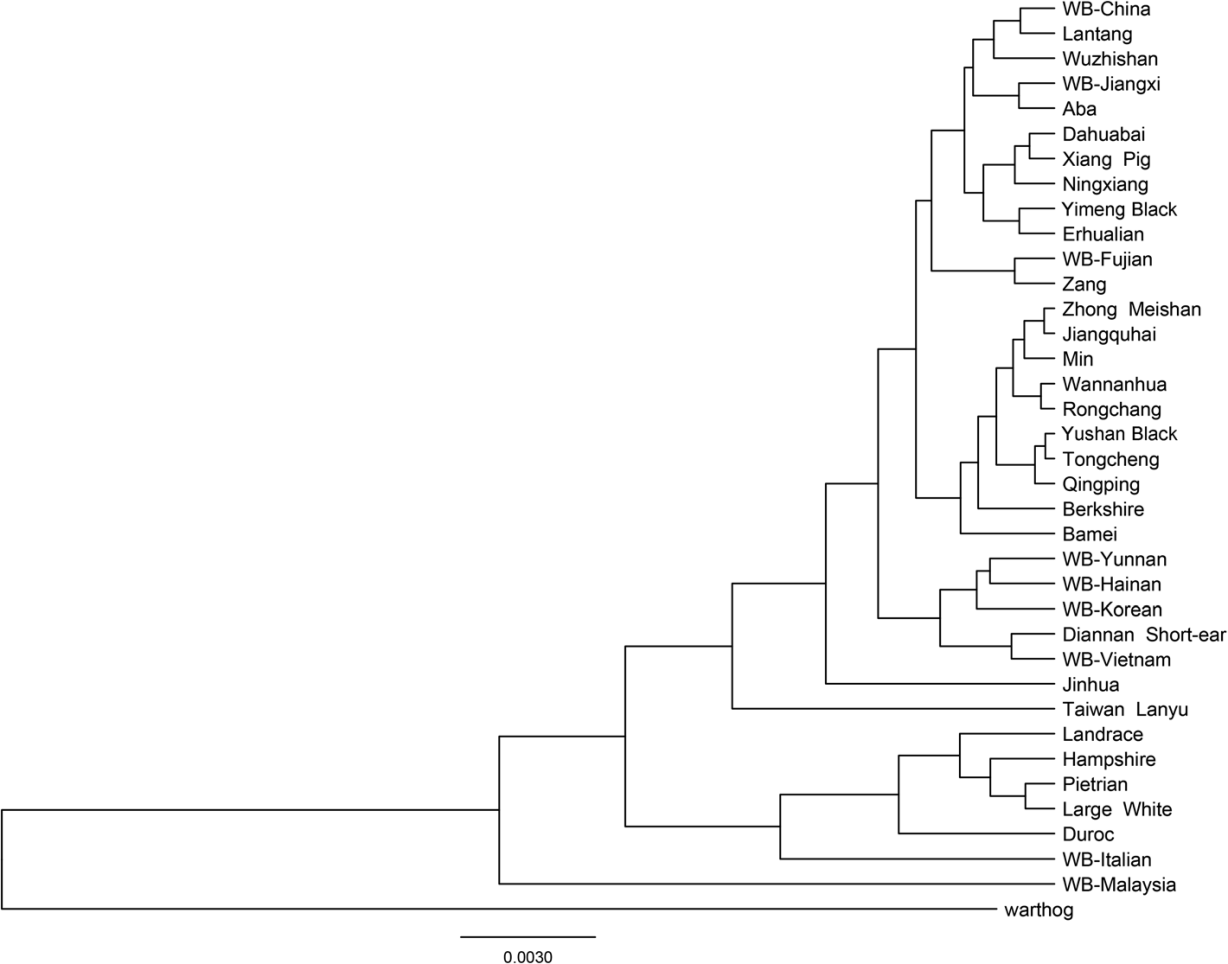
Consensus phylogenetic tree based on Bayesian calculation of 36 pig breeds by complete coding region of mtDNA sequences.

**Figure 2:**
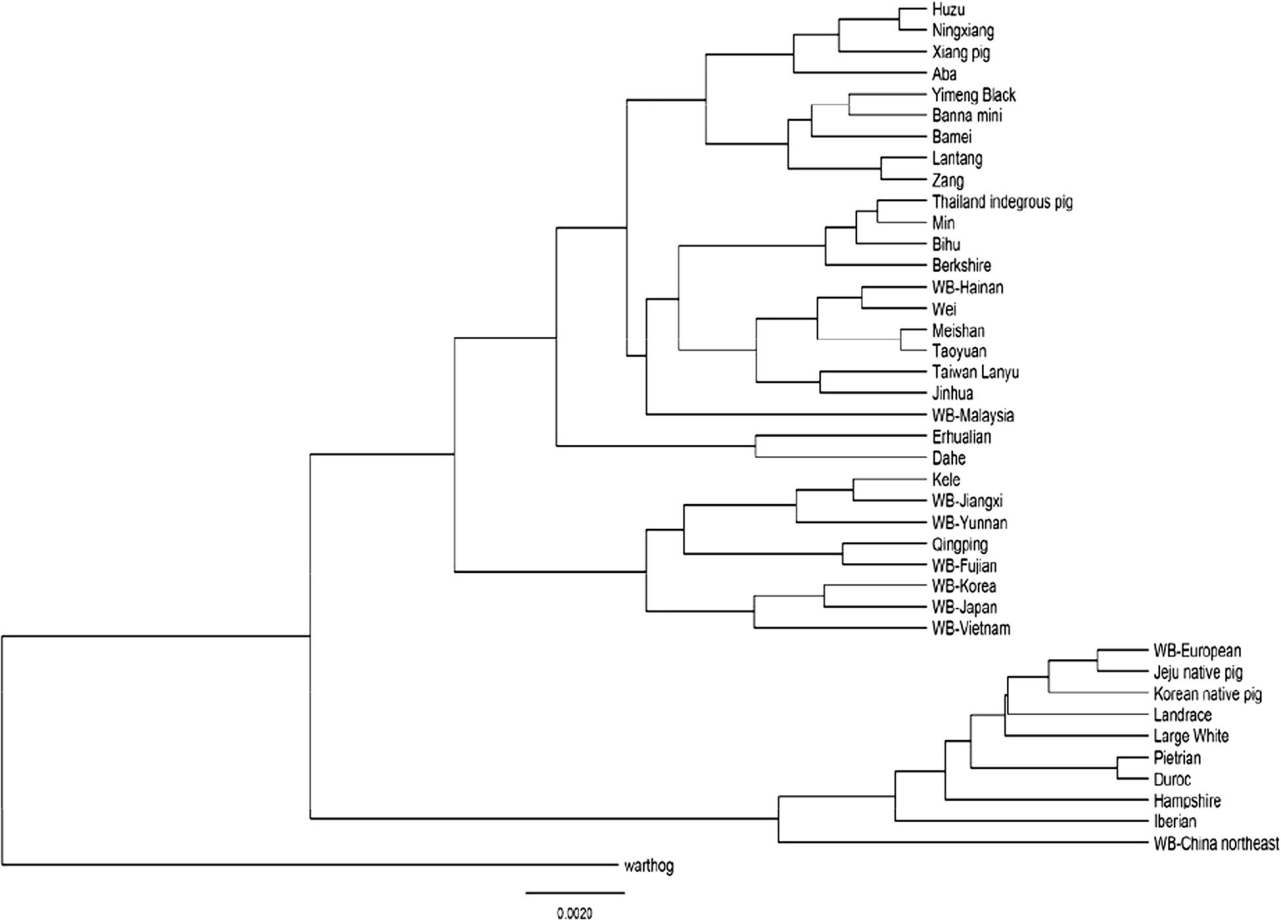
Consensus phylogenetic tree based on Bayesian calculation of 40 pig breeds by mtDNA D-loop sequences.

In this study, the complete mtDNA genomic sequences of Xiang pig, Lantang, Large White, Jinhua and Pietrain were sequenced and submitted to NCBI GenBank. And the other domestic pigs and wild boars sequences were downloaded from the GenBank for the analysis. We used the sequences both complete coding region mtDNA sequences and D-loop sequences for a contrast to analyze the origin and evolution of Chinese pigs. From the phylogenetic trees we could see that the two groups all showed the same two major clades: European clade and Asian clade, although there were some differences between them (Figure 1 and Figure 2) as some sequences were different from the two groups.

According to the complete coding region of mtDNA analysis, the WB-Malaysia fell outside the Eurasia domestic pigs and wild boars. The Lanyu pig showed distant genetic relationship with the Asian pigs. And European domestic pig breeds were showed to be closely related in their matrilines, the breed of Berkshire was separate from European breeds and clustered with Asian breeds. The information read from the complete coding region of mtDNA genome tree enabled us to conduct a phylogenomic analysis for wild boars and domestic pigs. The Lanyu and Jinhua were diverse more from other Chinese pigs. The WB-Yunnan, WB-Hainan, WB-Korean, Diannan Short-ear, WB-Vietnam were clustered into a subgroup. And the other two wild boar breeds of WB-Jiangxi and WB-Fujian were not clustered into the wild boar group, but they had near genetic distance to Aba and Zang respectively. WB-Fujian and Zang were clustered to a subclade and they were close with the South China and YZ region pigs. The other pig breeds all showed some regularity and relationship by their characteristics and geographic distribution. For example, Zhong Meishan, Jiangquhai and Min had a near genetic distance, and they were all characterized with black hair pigs, drop ear, drop abdomen and large body.

The results of nucleotide diversity calculated based on the maximum-likelihood method by complete coding region of mtDNA sequences were showed in Table 3. Pigs from the MK region had the most nucleotide diversity (0.01285 ± 0.00629) which contained the WB-Malaysia that was distant from both of the domestic pigs and wild boars (Figure 1). While the YZ group contained the most number of breeds, it shared the least number of nucleotide differences (10.43590) and the nucleotide diversity was 0.00068 ± 0.00013. The European pig clade also showed more nucleotide (0.00743 ± 0.00215) and average number of nucleotide differences (114.14286) which included Berkshire pig that was clustered into the Asian clade (Figure 1). It was the same as Lanyu pig which was belong to the South China but had a long genetic distance to the other pig breeds amongst the geography.

**Table 3:** Genetic diversity indices in 6 geographic groups from the complete coding region of mtDNA sequences

**Geographic definition**	**Breeds number**	**Nucleotide diversity**	**Average number of nucleotide differences**
North East Asia (NEA)	3	0.00629 ± 0.00203	96.66667
Yellow River Valley (YR)	2	0.00098 ± 0.00049	15.00000
Yangtze River Region (YZ)	13	0.00068 ± 0.00013	10.43590
Mekong Region (MR)	4	0.01285 ± 0.00629	197.50000
South China (SC)	6	0.00658 ± 0.00389	100.38095
European Country (EU)	7	0.00743 ± 0.00215	114.14286

While in the D-loop sequences phylogenetic tree, there were some differences within the clade compared to the complete coding region of mtDNA group although it also showed two significant main clades. Our findings clearly demonstrated that Chinese indigenous pigs were only recently diverged from each other and distinctly different from European-type pigs. It was surprised that two Korean native pigs and the WB-China northeast were clustered into the European clade. And WB-China northeast also had close genetic distance with the European clade. Within the Asian clade, all Asian domestic pig mtDNAs were further clustered into a subclade, with wild boars from this region intermingled. The domestic pigs of Kele and Qingping were included into the wild boar clade. The phylogenetic position of the Erhualian and Dahe fell outside the domestic pig clade containing Asian samples. The European breed of Berkshire was also clustered into the China clade as the result mentioned by complete coding region of mtDNA genomic calculation.

## Discussion

Our studies analyzed the phylogenetic relationship of Chinese pigs with other European and Asian domestic pigs and wild boars by both the complete coding region of mtDNA genome and the D-loop region calculation. The phylogenetic trees showed that the Berkshire belong to the Asian type, which was the same as other studies [27,36], although European domestic pig breeds are showed to be closely related in their matrilines. In our studies, the Large White was clustered into the European clade, it was different from other studies which showed the Large White was origin from Chinese pigs [27,28]. And there were only 8 single nucleotide polymorphisms (SNPs) between the Large White sequence we submitted to Genbank and the sequence downloaded from Genbank (NC_012095.1) except for the D-loop region. Two distinct mtDNA haplotypes (Asian and European) of the Large White breed have been reported, suggesting that cross-breeding between European and Asian pigs has occurred during the formation of this breed [12,20,37,38]. This also is expected for the Berkshire breed, as the study indicated that the Chinese breeds contributed significantly to the development of Berkshire [39].

There was a remarkable collection of phenotypic diversity between the Chinese indigenous pig breeds. The phylogenetic trees could not distinguish the subclades clearly by the geographic definition as some breeds might have some introgression during the breed development. And the present analysis attempted to measure the level of underlying genetic variation present within the complete coding region of mtDNA. The study based on the mtDNA D-loop found less genetic variation within Chinese breeds than within European breeds [40]. The Chinese, Japanese and Korean local breeds were separated in recent years and based on some limited factors; their classification may differ from the European type. The genetic difference of the Chinese Meishan and European breeds probably originated around 2,227 years ago [41]. By studying 48 local breeds, many China mainland breeds originated from Southeast Asia [27]. It was the same as our studies based on the complete coding region of mtDNA genome and D-loop region analysis.

From the phylogenetic tree based on complete coding region of mtDNA calculation, it was obvious that most of the Chinese pig breeds were origin from the wild boars distributed in the South China and the Yangtze River Region. It was similar to an Restriction Fragment Length Polymorphism (RFLP) study with mtDNA of local pig breeds sampled from south-eastern China [13]. Similarly, there was a suggestion that Chinese native pig breeds have a single origin [14]. Available zoo archaeological evidence has been interpreted to indicate that domestic pigs were prevalent in both northern and southern China by at least 8,000 B.P. [1]. By the D-loop region calculation, the Jeju native pig and Korean native pigs were clustered into the European clade. As the population was brought to the brink of extinction by the inflow of European exotic genes into the pig population on Jeju Island beginning about 50 years ago [27].

The Lanyu and Jinhua were classified as a new out-group and had a long genetic distance from other Chinese pigs. As the Lanyu belongs to a breed that is unique in the Taiwan islands, and there comes a phylogenetic relationship between the Lanyu and other small-ear strains [36]. This study showed that the Lanyu breed was an independent clade and was distant to any other Chinese pig breeds compared by the phylogenetic relationship of the D-loop region in the Lanyu and other pig breeds (Figure 1 and Figure 2). The Lanyu pig is of an independent branch but probably still belongs to the Asian type. This result implies that, the frequency of genetic exchange of the Lanyu with other pig breeds is low and they therefore do not have a close relationship [36]. Analysis showed that, the Lanyu could be an independent branch among the other pig breeds. The Chinese domestic populations were derived from multiple Asian ancestral origins whereas the European domestic populations represent a single ancestral European lineage [42].

The genetic diversity indices were higher than the normal regulation in some geographic groups. It was mainly determined by the isolation and introgression between other pig breeds. The Lanyu and WB-Malaysia were all isolated in the island and had a long genetic distance with other pigs. While the Berkshir was developed from the Chinese pigs and we also clustered it belonging to the European pigs.

## Conclusions

Compared the two mtDNA sequences calculations, the Chinese domestic pigs might be origin from the wild boars of Yangtze River Region and South China. Chinese pigs were involved in the development of Berkshire breeding. The cross-breeding between European and Asian pigs has occurred during the formation of Large White. This study does indicate that analysis of more animals using mtDNA will be informative in understanding the relationships between the breeds and introduce the ancient DNA of pigs to study the origination and evolution of the Chinese pigs.

## Abbreviations

mtDNA: Complete mitochondrial genomic sequences; WB: Wild boar; RFLP: Restriction Fragment Length Polymorphism; SNPs: Single nucleotide polymorphisms; NEA: North East Asia; YR: Yellow River Valley; YZ: Yangtze River Region; MK: Mekong Region; SC: South China; EU: European Country

## Competing interests

All of the authors declared there were not any financial competing interests in relation to this manuscript.

## Authors’ contributions

GY carried out the molecular genetic studies and drafted the manuscript. HX participated in the sequence alignment. JW carried out the PCR and sequencing. XZ conceived of the study, and participated in its design and coordination and helped to draft the manuscript. All authors read and approved the final manuscript.
